# A Tissue-Engineered Human Psoriatic Skin Model to Investigate the Implication of cAMP in Psoriasis: Differential Impacts of Cholera Toxin and Isoproterenol on cAMP Levels of the Epidermis

**DOI:** 10.3390/ijms21155215

**Published:** 2020-07-23

**Authors:** Mélissa Simard, Sophie Morin, Geneviève Rioux, Rachelle Séguin, Estelle Loing, Roxane Pouliot

**Affiliations:** 1 Centre de Recherche en Organogénèse Expérimentale de l’Université Laval/LOEX, Axe Médecine Régénératrice, Centre de Recherche du CHU de Québec—Université Laval, Québec, QC G1J 1Z4, Canada; melissa.simard.6@ulaval.ca (M.S.); sophie.morin.7@ulaval.ca (S.M.); genevieve.rioux.9@ulaval.ca (G.R.); rachelle.seguin.1@gmail.com (R.S.); 2 Faculté de Pharmacie, Université Laval, Québec, QC G1V 0A6, Canada; 3 IFF-Lucas Meyer Cosmetics, Québec, QC G1V 4M6, Canada; estelle.loing@lucasmeyercosmetics.com

**Keywords:** psoriasis, cyclic adenosine monophosphate, cholera toxin, isoproterenol, tissue engineering

## Abstract

Pathological and healthy skin models were reconstructed using similar culture conditions according to well-known tissue engineering protocols. For both models, cyclic nucleotide enhancers were used as additives to promote keratinocytes’ proliferation. Cholera toxin (CT) and isoproterenol (ISO), a beta-adrenergic agonist, are the most common cAMP stimulators recommended for cell culture. The aim of this study was to evaluate the impact of either CT or ISO on the pathological characteristics of the dermatosis while producing a psoriatic skin model. Healthy and psoriatic skin substitutes were produced according to the self-assembly method of tissue engineering, using culture media supplemented with either CT (10^−10^ M) or ISO (10^−6^ M). Psoriatic substitutes produced with CT exhibited a more pronounced psoriatic phenotype than those produced with ISO. Indeed, the psoriatic substitutes produced with CT had the thickest epidermis, as well as contained the most proliferating cells and the most altered expression of involucrin, filaggrin, and keratin 10. Of the four conditions under study, psoriatic substitutes produced with CT had the highest levels of cAMP and enhanced expression of adenylate cyclase 9. Taken together, these results suggest that high levels of cAMP are linked to a stronger psoriatic phenotype.

## 1. Introduction

Psoriasis is an autoimmune skin disease affecting 3% of the population worldwide and for which no cure currently exists [1]. Clinical manifestation of psoriasis is defined by the apparition of red plaques with white scales, which have detrimental consequences on patient’s quality of life [2,3]. Psoriasis patches can range from a few spots of dandruff-like scaling to major eruptions that cover large areas of the body. The histological hallmarks of psoriasis are a marked thickening of the epidermis, due to keratinocyte hyperproliferation, abnormal epidermal differentiation, and immune keratinocytes activation accompanied with immune cell infiltrate [4]. Consequently, the granular layer of the epidermis is reduced in thickness and the horny layer contains some undifferentiated keratinocytes, which still contain cell nuclei [5]. Furthermore, altered keratinocyte differentiation in psoriasis results at a molecular level in the deregulation of the epidermal differentiation marker proteins, such as involucrin (up-regulated), filaggrin (down-regulated), and keratin 10 (down-regulated) [6,7,8]. It is now well documented that the complex etiology of psoriasis involves interactions between environmental factors and complex genetic background [9]. However, the exact cause of psoriasis is still unknown, making it difficult to develop an effective treatment for the pathology [1].

Since the development of the first tissue-engineered skin model in 1978, by Green H. and colleagues, great deal of research has been conducted in this field, leading to the generation of highly reproducible and sophisticated skin models. In the last decade, the limits of tissue engineering have been constantly pushed back by the development of new emerging techniques, such as bio-printing [10,11]. However, the reconstruction of pathological skin models, such as psoriatic skin substitutes, remains challenging, especially since the exact causes of the pathology are still unknown. However, many psoriatic skin models were reconstructed in vitro using different tissue-engineered techniques [12]. According to Niehues et al., the self-assembly method is the one which leads to the reconstruction of substitutes faithfully mimicking psoriatic characteristics and which, consequently, offers an effective tool for the screening of new molecules [12,13,14]. Indeed, reconstructed psoriatic skin substitutes produced using cells from patients with psoriasis were shown to closely mimic the pathology, as they displayed enhanced epidermal thickness, hyperproliferative keratinocytes and disturbed epidermal differentiation. The morphology of reconstructed skin models is directly affected by culture conditions. While developing the first healthy tissue engineered model of skin in 1978, Green H. reported the importance of adding cAMP stimulator, such as cholera toxin (CT) or isoproterenol (ISO), to induce the proliferation of cells in culture [15]. Therefore, skin models are produced using culture media supplemented with a stimulator of the AC system, which is crucial for stimulating the colony growth of human keratinocytes and thus essential to obtain a fully differentiated epidermis [15,16,17,18,19,20].

**Figure 1:**
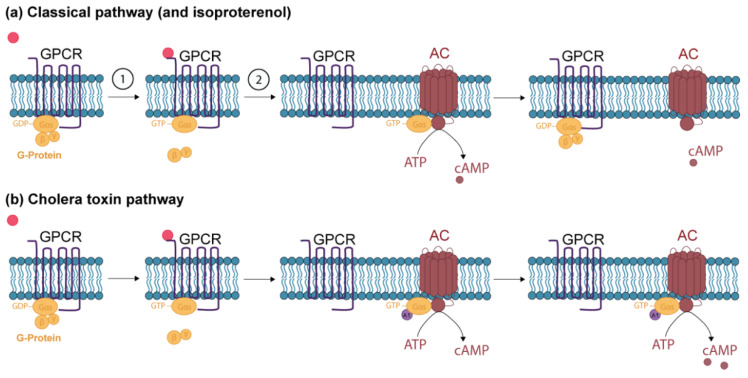
The cAMP signaling pathways. (**a**) 1: The first messenger (red) binds to the G protein-coupled receptor (GPCR). The receptor then changes conformation, leading to the replacement of GDP by GTP on the α subunit of the G protein and inducing the subsequent release of the α subunit from the β and γ subunits of the G protein [21,31]. 2: The α subunit binds to the catalytic domain of adenylate cyclase (AC). The α subunit can have inhibitory (α_i_) or stimulating (α_s_) properties, which will lead to the inhibition or stimulation of AC [16,32]. The activated AC will convert ATP into cAMP. Isoproterenol induces the conversion of ATP to cAMP following the classical pathway (**b**) The A1 peptide of cholera toxin (CT) (purple) binds to the complex composed of the GTP-α subunit of the G protein and AC and prevents the G protein from cleaving GTP to GDP, leading to a tremendous increase in cAMP levels [30].

The cAMP signaling pathway is composed of the first messenger, the G protein-coupled receptor (GPCR), the adenylate cyclase enzyme (AC) and the cAMP-degrading enzyme (Figure 1a). The epidermis contains four independent receptor AC systems: the β-adrenergic, prostaglandin E, adenosine, and histamine receptors [21,22,23]. The cAMP mainly acts as a second messenger, by stimulating different proteins, such as protein kinase A (PKA) and exchange protein directly activated by cAMP (EPAC). PKA and EPAC are then able to modify cell activity by phosphorylating diverse proteins [18]. The action of cAMP ends upon its hydrolysis by phosphodiesterases [24,25,26]. CT and ISO induce the production of cAMP following a different pathway. ISO is an analogue to epinephrine and binds exclusively to β-adrenergic receptors, which then stimulates cAMP production following the classical pathway (Figure 1a) [27,28]. CT binds to ganglioside GM1 receptors on epithelial cells, which triggers endocytosis, thus transporting the receptors to the endoplasmic reticulum (ER). From the lumen of the ER, the A1 peptide of the CT is transported into the cytoplasm, where it prevents the G protein from cleaving GTP to GDP, leading to a tremendous increase in cAMP levels (Figure 1b) [16,29,30]. 

The impact of different cAMP inducers (CT and ISO) on the morphology of psoriatic skin models has not yet been investigated, whereas it should be chosen with much consideration, especially since controversial results have been found regarding the involvement of cAMP in psoriasis. Indeed, Voorhes et al. suggested in the seventies that an alteration in cAMP levels could be involved in psoriasis [33]. Moreover, a few studies have reported lower levels of cAMP in psoriatic skin, suggesting that low levels of cAMP were linked with enhanced cell proliferation and thus contrasting with finding of Green H [33,34,35,36,37]. The aim of the present study was to therefore compare the use of both cAMP enhancers (CT or ISO) in the reconstruction of psoriatic skin substitutes to establish which one would lead to the better psoriatic phenotype, with traits, such as cell hyperproliferation and disturbed cell differentiation. The cAMP signaling pathway was then investigated in detail to demystify cAMP levels, as well as the activity of various agents of the AC system, in the pathology of psoriasis. Therefore, the current study brings new insights to the long-standing debate as to whether cAMP is increased or decreased in psoriasis.

## 2. Results

### 2.1. Differential Impact of CT and ISO on the Psoriatic Skin Substitute Phenotype: Epidermal Hyperproliferation

**Figure 2:**
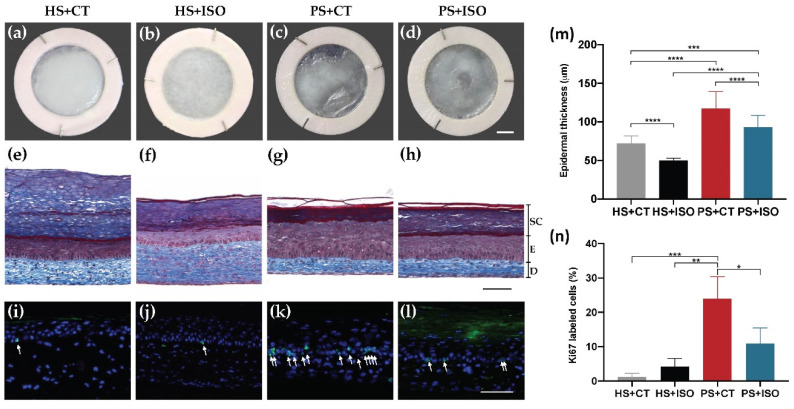
Morphology and epidermal proliferation of healthy substitutes (HS) and psoriatic substitutes (PS) produced with cholera toxin (+CT) or isoproterenol (+ISO). (**a**–**d**) Skin substitute macroscopic aspects; (**e**–**h**) Masson’s trichrome staining of skin substitute histological sections (D: Dermis, E: Epidermis living layers, SC: *Stratum corneum*); (**i**–**l**) Ki67 immunofluorescence (green) detecting keratinocytes in proliferation. White arrows indicate Ki67-positive cells. Nuclei were stained with Hoechst (blue); (**m**) quantification of the thickness of the epidermal living layer (designated as E in panels (**e**–**h**) measured from Masson’s trichrome staining using the ImageJ software; (**n**) ratio of Ki67 positive cells to the number of total keratinocytes in the basal layer. Scale bars: (**a**–**d**) 5 mm; (**e**–**h**) 100 μm. Data presented are the means +SD (*N* = 2 donors per condition, *n* = 3 skin substitutes per donor). The statistical significance was determined using one-way ANOVA followed by a Tukey’s post-hoc test. (* *p*-value < 0.05; ** *p*-value < 0.01; *** *p*-value < 0.001; **** *p*-value < 0.0001).

According to the macroscopic aspect of the skin substitutes, the epidermis of psoriatic substitutes reconstructed using culture media supplemented with CT (PS+CT) and the epidermis of psoriatic substitutes reconstructed using ISO (PS+ISO) were more disorganized since they displayed a less opaque and uniform surface than those of healthy substitutes (HS+CT) and (HS+ISO) (Figure 2c,d vs. Figure 2a,b). Moreover, both PS+CT and PS+ISO had a significantly thicker epidermis than their respective counterparts, showing higher proliferation of the keratinocytes (Figure 2g,h vs. Figure 2e,f). Hematoxylin and eosin staining of the skin substitutes are presented in Figure S1. This enhanced proliferation was confirmed with Ki67 immunofluorescence showing more basal keratinocytes in cellular division in PS than in HS (Figure 2k,l vs. Figure 2i,j). However, keratinocytes from PS+ISO were not as hyperproliferative as those in PS+CT. In fact, the epidermis of PS+ISO was not as thick as that of PS+CT (Figure 2m). The Ki67 staining confirmed these results (Figure 2n). Taken together, these results showed that both PS+CT and PS+ISO displayed higher levels of epidermal proliferation than their healthy counterparts HS+CT and HS+ISO, respectively. Moreover, the hyperproliferation was greater in the epidermis of PS+CT than PS+ISO.

### 2.2. Differential Impact of CT and ISO on the Psoriatic Skin Substitute Phenotype: Disturbed Epidermal Differentiation

**Figure 3:**
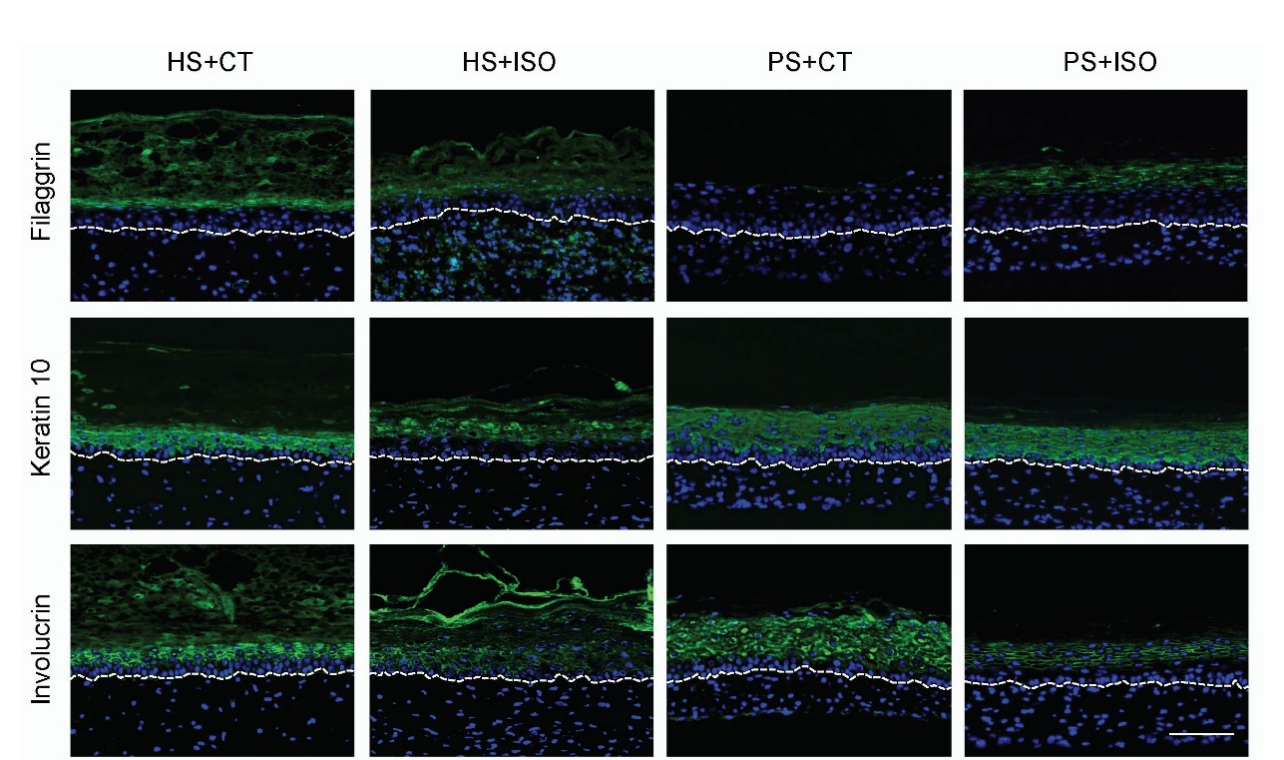
Expression of epidermal differentiation markers (green) in healthy substitutes (HS) and psoriatic substitutes (PS) produced with cholera toxin (+CT) or isoproterenol (+ISO) (*N* = 2 donors per condition, *n* = 3 skin substitutes per donor). The nuclei were stained with Hoechst (blue). The dotted line indicates the dermo-epidermal junction. Scale bar: 100 μm.

The expression of differentiation markers was strongly altered in PS+CT compared with HS+CT, thus showing a psoriatic phenotype such as expected. Indeed, the late differentiation markers filaggrin and keratin 10 were both down-regulated while the expression of the early differentiation marker involucrin was up-regulated (Figure 3). On the other hand, the effects of ISO on the expression of the differentiation markers were not as conclusive as for CT. In fact, the expression of filaggrin and keratin 10 for PS+ISO was down-regulated but not as much as for PS+CT compared with their respective counterparts. The involucrin staining was even less conclusive, since its expression in PS+ISO appeared less intense than the other conditions. According to these results, PS produced with ISO displayed a complete epidermal differentiation similar to what was observed for HS, since late differentiation markers are expressed. Therefore, epidermal differentiation in PS+ISO did not properly mimic the disturbed epidermal differentiation characteristics of psoriasis, which further entail that ISO is not a suitable cAMP stimulator to produce psoriatic skin substitutes.

### 2.3. Levels of cAMP in the Epidermis of Psoriatic Substitutes Produced with either CT or ISO

**Figure 4:**
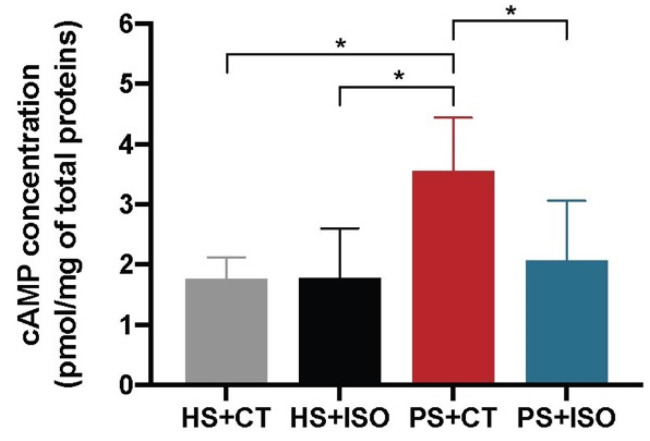
cAMP levels in the epidermis of healthy substitutes (HS) and psoriatic substitutes (PS) produced with either cholera toxin (+CT) or isoproterenol (+ISO). Data presented are the means +/− SD (*N* = 2 donors per condition, *n* = 2 skin substitutes per donor). The statistical significance was determined using one-way ANOVA followed by a Tukey’s post-hoc test. (* *p*-value < 0.05).

The levels of cAMP in the epidermis of HS+CT and HS+ISO were approximately the same (Figure 4.) The two cAMP enhancers, therefore, have similar effects on the AC activity of HS. The level of cAMP for PS+CT was significantly higher than for the other three conditions, revealing a greater capacity of CT to stimulate the production of cAMP.

### 2.4. Identification of Isoforms of cAMP-Related Protein Found in the Skin

Gene profiling on microarray was exploited to examine which AC were expressed in the skin substitutes (Table 1). Among the 10 AC isoform genes (*ADCY1-10*), only *ADCY3*, *ADCY7*, and *ADCY9* displayed a linear signal over 100 and were therefore identified as expressed genes in the HS+CT and PS+CT. Interestingly, the levels of expression of the three *ADCY* genes were higher (2-fold) in PS+CT than in HS+CT. Furthermore, expression of the *ADRB2* gene encoding for the beta-2 adrenergic receptor was also detected in both HS+CT and PS+CT. The expression of *ADRB2* was decreased (0.5-fold) in PS+CT.

### 2.5. Levels of cAMP-Related Proteins in the Epidermis of Psoriatic Substitutes Produced with Either CT or ISO

**Figure 5:**
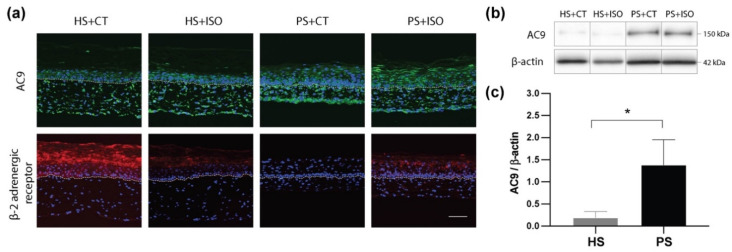
(**a**) Expression of adenylate cyclase 9 (AC9) (green) and β2-adrenergic receptor (red) in healthy substitutes (HS) and psoriatic substitutes (PS) produced with cholera toxin (+CT) or isoproterenol (+ISO). The nuclei were stained with Hoechst (blue). The dotted line indicates the dermo-epidermal junction. Scale bar: 100 μm. (**b**) Ten micrograms of total protein from skin substitute epidermis were analyzed by immunoblot for the presence of adenylate cyclase 9 (AC9). β-actin was used to control equal loading. (*N* = 2 donors per condition; *n* = 2 skin substitutes per donor). One representative immunoblot is shown. (**c**) Densitometric analyses of the immunoblot from panel (**b**) (*N* = 2 donors per condition; *n* = 2 skin substitutes per donor). Data from healthy or psoriatic substitutes were combined irrespective of treatment regime. The statistical significance was determined using an unpaired *t*-test (* *p*-value < 0.05).

Immunofluorescence staining was performed to validate the epidermal levels of AC9 and beta-2 adrenergic receptor, which are encoded by *ADCY3, ADCY7*, *ADCY9*, and *ADRB2* genes, respectively, as well as to compare the impact of CT and ISO on those protein levels (Figure 5a). Based on the immunofluorescence staining, the AC9 was found in the cells of both the dermis and the epidermis of the skin substitutes. Moreover, AC9 levels seemed up-regulated in psoriatic substitutes (PS+CT and PS+ISO) as compared with healthy ones. On the other hand, the β2-adrenergic receptor was found predominantly in the epidermis. Interestingly, high levels of the β2-adrenergic receptor were detected in HS+CT, while low levels were found in HS+ISO and it was not detected in PS+ISO. These results therefore support the conclusion that the levels of β2-adrenergic receptors are decreased in psoriatic skin.

Western blot analyses were next conducted to validate the levels of AC9 in the epidermis of the skin substitutes, since immunofluorescence staining of these proteins was used for qualitative purposes. AC9 was easily detected in the epidermis of all conditions, with a higher prevalence in PS+CT and PS+ISO (Figure 5b,c). AC3 and AC7 were not detected in the epidermis of either healthy nor psoriatic skin substitutes under our experimental conditions. 

## 3. Discussion

Culture media used to generate tissue-engineered skin substitutes are supplemented with a cAMP stimulator, which ensures epithelial cell proliferation [15,17]. In the present study, it was shown that even if the psoriatic skin models reconstructed with either CT or ISO displayed pathological features of psoriasis, CT remains more effective for stimulating the cells towards a psoriatic phenotype with hyperproliferative epidermal cells. Psoriatic substitutes produced with CT also displayed higher cAMP levels, as well as higher AC9 expression, than other conditions. These results show that higher cAMP levels would be associated with a stronger psoriatic phenotype, such as epidermal hyperproliferation and altered epidermal differentiation, therefore supporting studies in which cAMP levels are found to be increased in psoriatic skin.

Our data suggest that, in the presence of healthy keratinocytes, both cAMP enhancers (CT and ISO) stimulate the production of cAMP in the same manner, although the concentration of CT used in the experiment was lower than the concentration of ISO. Therefore, these results are in accordance with previous studies, which have reported that even if CT and ISO are both cAMP stimulators of epidermal cells, CT is better than ISO for the improvement of keratinocyte growth since it may increase the overall growth rate of epidermal cells by reducing the doubling time of cells [15]. In vitro studies reported by Green et al. have shown that using 10^−6^ M of isoproterenol produced an increase in keratinocyte colony size but had less effect on cAMP levels than 10^−6^ M of CT. Indeed, CT was the strongest agent in affecting cAMP concentration among the four different cAMP stimulators tested [15]. Moreover, these results support the use of both cAMP inducers, at appropriate concentrations, when producing healthy reconstructed skin models, and are in accord with results reported by Cortez Ghio et al. [38]. Isoproterenol (ISO) is therefore a good candidate for replacing CT since it presents several advantages regarding safety and regulations [38]. Indeed, CT is known to be potentially toxic and therefore requires more precautions in its manipulation [39,40]. 

In psoriatic substitutes, CT induced a stronger psoriatic phenotype, as well as higher levels of cAMP, than ISO when using the same concentration of each inducer as for the healthy models. This suggests that CT would be a better cAMP stimulator for lesional cells and that unlike for healthy substitutes, ISO is not recommended to stimulate cAMP in lesional cells. This incapacity of ISO to stimulate cAMP production in psoriatic substitutes could be attributed to a beta-adrenergic defect in psoriatic skin. Although Archer et al. once reported that psoriasis is not associated with impaired beta-adrenergic reactivity [41], most studies have since reported a decrease in responsiveness to beta-adrenergic stimuli, such as isoproterenol, in the stimulation of cAMP production in psoriatic tissues [16,30,42,43,44]. This decreased responsiveness would be linked to a lower expression of the beta-adrenergic receptor in psoriatic skin, which was confirmed in the present study with immunofluorescence staining. Different beta-2-adrenergic receptor polymorphisms may also contribute to the pathogenesis [30,45]. This defect of the beta-2-adrenergic receptor in psoriatic skin does not affect cAMP stimulation by CT, since the CT mechanism of action is based on direct activation of AC9. Finally, in 1978, Das et al. reported that the topical application of 0.1% isoproterenol sulphate mixed with white Vaseline and applied to the psoriatic plaques of twelve patients induced a significant decrease in the scaliness and cell turnover of the treated psoriatic skin [46]. Even if no other literature was found on a possible commercial development of this formulation, it appears that isoproterenol could be a potential topical or systemic treatment for psoriatic patients.

In the present study, a stronger psoriatic phenotype and higher levels of cAMP were found in psoriatic substitutes as compared with healthy substitutes, especially with CT, for which the difference was significant. These results therefore imply that higher levels of cAMP would be found in psoriatic skin, which is in contrast to the levels of cAMP monitored in psoriatic skin in the late seventies [33]. However, these results are in accordance with studies on the effects of CT on psoriasis in vivo, in which it was revealed that the psoriatic epidermis accumulates much more cAMP than uninvolved body regions or normal human epidermis [18,21]. According to the present study, the higher levels of cAMP found in psoriatic epidermis could be attributed to enhanced AC9 expression. AC9 is mostly expressed in the brain, spinal cord, liver, heart, lung, kidney, muscle, and adrenal gland, but little is known about its localization in the skin, particularly in the epidermis [24,47,48]. Finally, enhanced levels of cAMP in psoriatic skin are more consistent with the levels of downstream mediators identified in the literature during the past decade [49,50,51]. Indeed, it is relatively well established that psoriatic skin displays increased levels of phosphorylated CREB, which controls cellular functions, such as the regulation of gene expression [52].

In summary, our study showed that, although both cAMP inducers stimulate the production of psoriatic skin substitutes displaying hyperproliferating epidermis and disturbed epidermal differentiation, CT induced a stronger psoriatic phenotype than ISO. Moreover, enhanced cAMP levels were found in the epidermis of our psoriatic substitutes, which could be attributed to enhanced expression of AC9 in psoriatic skin. Therefore, our study answers a highly controversial question, suggesting that cAMP levels are increased in psoriatic skin. Finally, cAMP levels were higher in the CT psoriatic substitutes than in the ISO psoriatic substitutes, confirming a defect of the beta2-adrenergic receptors in psoriatic skin. This study is of particular interest since to our knowledge it is one of the first to determine the concentrations of cAMP in a psoriatic reconstructed skin model produced by tissue engineering.

## 4. Materials and Methods 

### 4.1. Cell Culture

This study was conducted in agreement with the Helsinki Declaration and performed under the guidelines of the Research Ethics Committee of the Centre de recherche du CHU de Québec – Université Laval. All patients were given adequate information for providing written consent. Two different patients with psoriasis aged 46 and 49 years old, respectively, were recruited. Six-millimeter punch biopsies were taken from psoriatic skin. As for the healthy skin substitutes, biopsies were obtained from healthy donors during breast reduction surgeries. Healthy donors were Caucasian females aged 46 and 49 years old. Cells were extracted according to the method based on thermolysin, trypsin, and collagenase digestions described elsewhere [53].

### 4.2. Skin Substitute Production

All skin substitutes were produced according to the self-assembly method of tissue engineering [13]. Briefly, fibroblasts at passage 6 were seeded in 6-well plates at 0.12 × 10^6^ cells per well. Fibroblasts were cultured in Dulbecco’s modified Eagle’s medium (DME) (Gibco, Life Technologies, New York, NY, USA) supplemented with 10% Fetal Calf premium Serum (FCS) (Wiscent, Inc., St-Bruno, QC, Canada), 50 μg/mL ascorbate acid (Sigma, Oakville, ON, Canada) and antibiotics; 60 μg/mL penicillin G (Sigma, Oakville, ON, Canada) and 25 μg/mL gentamicin (Schering, Pointe-Claire, QC, Canada). After 28 days, dermal cells formed sheets that were superimposed and cultured for another 2 d. After that, keratinocytes at passage 2 were seeded at 1.2 × 10^6^ cells upon each tissue sheet to form the epidermal layer. Another 7 days of culture allowed the epidermal cells to grow, and then each substitute was raised to the air-liquid interface and cultured for a total of 21 days. Keratinocytes were cultured in DME mixed with Ham’s F12 medium (3:1) (DME-HAM) (Gibco, Life Technologies, New York, NY, USA) supplemented with 5% FetalClone II serum (Hyclone, Logan, UT, US), 5 μg/mL insulin (Sigma, Oakville, ON, Canada), 0.4 μg/mL hydrocortisone (Galenova, St-Hyacinthe, QC, Canada), 10 ng/mL human epidermal growth factor (EGF) (Ango Inc, San Ramon, CA, USA), 60 μg/mL penicillin, and 25 μg/mL gentamicin. Keratinocyte culture media were also supplemented with either 10^−10^ M cholera toxin (MP Biomedicals, Montreal, QC, Canada) or 10^−6^ M isoproterenol (rISO; Sigma Aldrich, St. Louis, MO, USA). 

### 4.3. Histological Analyses

Biopsies of skin substitutes were analyzed by histological and immunohistochemical methods after 21 days of culture at the air-liquid interface. For living epidermal thickness analyses, two biopsies of each condition were fixed in Histochoice^®^ (AMRESCO, Inc., Solon, OH, USA) and embedded in paraffin wax. After that, deparaffinized 5 µm tissue sections were cut and stained with Masson’s Trichrome. The thickness of the living epidermis was obtained by measures made with Image J software (National Institutes of Health, USA, http://imagej.nih.gov/ij). Ten measurements in three different parts of each skin substitute were used to compare the living epidermis thickness between skin substitutes. 

### 4.4. Indirect Immunofluorescence on Frozen Tissues

For immunofluorescence staining, tissues were embedded in Tissue-Tek O.C.T Compound (Sakura Finetek, CA, USA) and 6 µm thick sections were fixed in acetone at −20 °C before staining. The following antibodies were used and incubated in a dark room at room temperature for 45 min: rabbit anti-involucrin (Abcam, Cambridge, MA, USA), rabbit anti-filaggrin (Abcam, Cambridge, MA, USA), rabbit anti-keratin 10 (Abcam, Cambridge, MA, USA), mouse anti-Ki67 IgG1 (BD Biosciences, CA, USA), rabbit anti-AC9 (ab191423, Abcam, Cambridge, MA, USA) and rabbit anti-beta 2 adrenergic receptor (ab61778, Abcam, Cambridge, MA, USA). Tissues were then incubated with Alexa Fluor 488 donkey anti-rabbit IgG or Alexa Fluor 594 goat anti-mouse IgG (1:1600, Thermofisher Scientific, CA, USA) for 30 min also at room temperature. Nuclear counter staining using DAPI (SouthernBiotech, AL, USA) was then effected on different samples. Each tissue was observed using a Zeiss Axio Imager M2 microscope with an AxioCam ICc1 camera. The quantification of immunofluorescence staining was performed by densitometry using ImageJ software (from Wayne Rasband, National Institute of Health (NIH), USA).

### 4.5. Cyclic AMP Competitive ELISA Kit on Frozen Tissues

After 63 days of cell culture to prepare the reconstructed skin substitutes, the epidermis was mechanically separated from the dermis using scalpel and forceps, and samples were quick-frozen in liquid nitrogen to preserve the tissue integrity. Tissues were crushed in a Safe-Lock 2.0 mL Eppendorf tube (ATS Scientific, Inc., Burlington, ON, Canada) with two 6 mm stainless ball using a Cryomill MM400 (Retsch^®^, Newtown, PA, USA). The levels of cAMP were assayed using a Cyclic AMP Competitive ELISA Kit (ThermoFisher Scientific, Vienna, Austria). The non-acetylated version of the ELISA Kit was used following the protocol provided by the manufacturer, and 400 μg of cell lysate was used in the test. Total protein concentrations were determined using a BCA Protein Assay Kit according to the manufacturer’s instructions (Thermo Scientific, Rockford, IL, USA). 

### 4.6. Western Blots

Western blots were conducted using the following primary antibodies: rabbit anti-AC3 (ab14778, Abcam, Cambridge, MA, USA), rabbit anti-AC7 (ab14782, Abcam, Cambridge, MA, USA), and rabbit anti-AC9 (ab191423, Abcam, Cambridge, MA, USA). The secondary antibodies used were: goat anti-rabbit HRP labeled (1:60,000, Jackson ImmunoResearch Laboratories, Inc., West Grove, PA, USA); and goat anti-mouse HRP labeled (1:60,000, Jackson ImmunoResearch Laboratories, Inc., West Grove, PA, USA). Proteins of interest were detected using ECL Prime Western Blotting Detection Reagent (GE Healthcare, Little Chalfont, UK) and Fusion Fx7 imager (MBI Lab Equipment, Kirkland, QC, Canada). Quantification of immunoblots was performed by densitometry using ImageJ software (from Wayne Rasband, National Institute of Health (NIH), USA).

### 4.7. Gene Expression Profiling

Total RNA was isolated from skin substitutes using the RNeasy Mini Kit (QIAGEN, Toronto, ON, Canada) and its quality determined (2100 Bioanalyzer, Agilent Technologies, Mississauga, ON, Canada) as described [54]. Labeling of Cyanine 3-CTP-labeled targets, their hybridization on a G4851A SurePrint G3 Human GE 8 × 60K array slide (Agilent Technologies, Santa-Clara, CA, USA), and data acquisition and analyses were all performed as previously reported [54]. 

## Figures and Tables

**Table 1:** Linear signals and fold change for *ADCY1-10* and *ADRB2* genes between healthy and psoriatic substitutes produced with CT.

Gene Symbol	Gene Name	Linear Signal HS+CT	Linear Signal PS+CT	Fold Change PS/HS	*p*-Value
*ADCY1*	Adenylate cyclase type 1	6.688	11.806	1.765	0.0628
*ADCY2*	Adenylate cyclase type 2	2.953	3.933	1.332	0.0942
*ADCY3*	Adenylate cyclase type 3	1714.868	3504.636	2.044	0.0359
*ADCY4*	Adenylate cyclase type 4	59.773	43.994	0.736	0.1634
*ADCY5*	Adenylate cyclase type 5	7.595	5.103	0.672	0.2416
*ADCY6*	Adenylate cyclase type 6	55.333	127.404	2.302	0.2256
*ADCY7*	Adenylate cyclase type 7	275.730	614.908	2.230	0.0446
*ADCY8*	Adenylate cyclase type 8	5.560	4.526	0.814	0.5801
*ADCY9*	Adenylate cyclase type 9	228.869	549.184	2.400	0.0471
*ADCY10*	Adenylate cyclase type 10	7.348	5.591	1.286	0.1556
*ADRB2*	Beta-2 adrenergic receptor	338.779	160.034	0.472	0.0487

## Supplementary Materials

Supplementary Materials can be found at https://www.mdpi.com/1422-0067/21/15/5215/s1.

## Abbreviations

CT	Cholera toxin
ISO	Isoproterenol
AC	Adenylate cyclase enzyme
GPCR	G protein-coupled receptor
PKA	Protein kinase A
ER	Endoplasmic reticulum
HS	Healthy substitutes
PS	Psoriatic substitutes

## References

[1] Boehncke W.-H., Schön M.P. (2015). Psoriasis. Lancet.

[2] Lowes M.A., Bowcock A.M., Krueger J.G. (2007). Pathogenesis and therapy of psoriasis. Nature.

[3] Krueger G., Koo J., Lebwohl M., Menter A., Stern R.S., Rolstad T. (2001). The impact of psoriasis on quality of life: Results of a 1998 National Psoriasis Foundation patient-membership survey. Arch. Dermatol..

[4] Mak R.K., Hundhausen C., Nestle F.O. (2009). Progress in understanding the immunopathogenesis of psoriasis. Actas Dermo Sifiliogr..

[5] Schön M.P., Boehncke W.-H. (2005). Psoriasis. N. Engl. J. Med..

[6] Chen J.-Q., Man X.-Y., Li W., Zhou J., Landeck L., Cai S.-Q., Zheng M. (2013). Regulation of Involucrin in Psoriatic Epidermal Keratinocytes: The Roles of ERK1/2 and GSK-3β. Cell Biochem. Biophys..

[7] Wolf R., Orion E., Ruocco E., Ruocco V. (2012). Abnormal epidermal barrier in the pathogenesis of psoriasis. Clin. Dermatol..

[8] Danso M., Boiten W., Van Drongelen V., Meijling K.G., Gooris G., El Ghalbzouri A., Absalah S., Vreeken R.J., Kezic S., Van Smeden J. (2017). Altered expression of epidermal lipid bio-synthesis enzymes in atopic dermatitis skin is accompanied by changes in stratum corneum lipid composition. J. Dermatol. Sci..

[9] Benhadou F., Mintoff D., Del Marmol V. (2019). Psoriasis: Keratinocytes or Immune Cells—Which Is the Trigger?. Dermatology.

[10] Shi Y., Xing T.L., Zhang H.B., Yin R.X., Yang S.M., Wei J., Zhang W.J. (2018). Tyrosinase-doped bioink for 3D bioprinting of living skin constructs. Biomed. Mater..

[11] Lee V., Singh G., Trasatti J.P., Bjornsson C., Xu X., Tran T.N., Yoo S.-S., Dai G., Karande P. (2014). Design and Fabrication of Human Skin by Three-Dimensional Bioprinting. Tissue Eng. Part C Methods.

[12] Desmet E., Ramadhas A., Lambert J., Van Gele M. (2017). In vitro psoriasis models with focus on reconstructed skin models as promising tools in psoriasis research. Exp. Biol. Med..

[13] Jean J., Lapointe M., Soucy J., Pouliot R. (2009). Development of an in vitro psoriatic skin model by tissue engineering. J. Dermatol. Sci..

[14] Niehues H., van den Bogaard E.H. (2018). Past, present and future of in vitro 3D reconstructed inflammatory skin models to study psoriasis. Exp. Dermatol..

[15] Green H. (1978). Cyclic AMP in relation to proliferation of the Epidermal cell: A new view. Cell.

[16] Tamura T., Takahashi H., Ishida-Yamamoto A., Hashimoto Y., Iizuka H. (1998). Functional alteration of guanine nucleotide binding proteins (Gs and Gi) in psoriatic epidermis. J. Dermatol. Sci..

[17] Okada N., Kitano Y., Ichihara K. (1982). Effects of Cholera Toxin on Proliferation of Cultured Human Keratinocytes in Relation to Intracellular Cyclic AMP Levels. J. Investig. Dermatol..

[18] Takahashi H., Honma M., Miyauchi Y., Nakamura S., Ishida-Yamamoto A., Iizuka H. (2004). Cyclic AMP differentially regulates cell proliferation of normal human keratinocytes through ERK activation depending on the expression pattern of B-Raf. Arch. Dermatol. Res..

[19] Duque-Fernandez A., Gauthier L., Simard M., Jean J., Gendreau I., Morin A., Soucy J., Auger M., Pouliot R. (2016). A 3D-psoriatic skin model for dermatological testing: The impact of culture conditions. Biochem. Biophys. Rep..

[20] Löwa A., Vogt A., Kaessmeyer S., Hedtrich S. (2018). Generation of full-thickness skin equivalents using hair follicle-derived primary human keratinocytes and fibroblasts. J. Tissue Eng. Regen. Med..

[21] Iizuka H., Matsuo S., Tamura T., Ohkuma N. (1988). Increased Cholera Toxin-, and Forskolin-induced Cyclic AMP Accumulations in Psoriatic Involved Versus Uninvolved or Normal Human Epidermis. J. Investig. Dermatol..

[22] Lee T.P., Busse W.W., Reed C.E. (1974). Epidermal adenyl cyclase of human and mouse. A study of the atopic state. J. Allergy Clin. Immunol..

[23] Andrés R.M., Terencio M.C., Arasa J., Payá M., Valcuende-Cavero F., Navalón P., Montesinos M.C. (2017). Adenosine A2A and A2B Receptors Differentially Modulate Keratinocyte Proliferation: Possible Deregulation in Psoriatic Epidermis. J. Investig. Dermatol..

[24] Adachi K., Iizuka H., Halprin K.M., Levine V. (1977). Specific refractoriness of adenylate cyclase in skin to epinephrine, prostaglandin E, histamine and AMP. Biochim. Biophys. Acta (BBA) Gen. Subj..

[25] Stratakis C.A. (2012). Cyclic AMP, Protein Kinase A, and Phosphodiesterases: Proceedings of an International Workshop. Horm. Metab. Res..

[26] Sakkas L.I., Mavropoulos A., Bogdanos D.P. (2017). Phosphodiesterase 4 Inhibitors in Immune-mediated Diseases: Mode of Action, Clinical Applications, Current and Future Perspectives. Curr. Med. Chem..

[27] Warne A., Moukhametzianov R., Baker J.G., Nehmé R., Edwards P.C., Leslie A.G.W., Schertler G.F.X., Tate C.G. (2011). The structural basis for agonist and partial agonist action on a β1-adrenergic receptor. Nature.

[28] Ji Y., Chen S.-Y., Li K., Xiao X., Zheng S., Xu T. (2013). The role of β-adrenergic receptor signaling in the proliferation of hemangioma-derived endothelial cells. Cell Div..

[29] Androutsellis-Theotokis A., Walbridge S., Park D.M., Lonser R.R., McKay R.D.G. (2010). Cholera Toxin Regulates a Signaling Pathway Critical for the Expansion of Neural Stem Cell Cultures from the Fetal and Adult Rodent Brains. PLoS ONE.

[30] Sivamani R.K., Lam S.T., Isseroff R.R. (2007). Beta Adrenergic Receptors in Keratinocytes. Dermatol. Clin..

[31] Choi E.J., Toscano W.A. (1988). Modulation of adenylate cyclase in human keratinocytes by protein kinase C. J. Biol. Chem..

[32] Cumbay M.G., Watts V.J. (2004). Novel Regulatory Properties of Human Type 9 Adenylate Cyclase. J. Pharmacol. Exp. Ther..

[33] Voorhees J.J., Duell E.A. (1971). Psoriasis as a possible defect of the adenyl cyclase-cyclic AMP cascade. A defective chalone mechanism?. Arch. Dermatol..

[34] Bass L.J., Powell J.A., Voorhees J.J., Duell E.A., Harrell E.R. (1972). The Cyclic Amp System in Normal and Psoriatic Epidermis. J. Investig. Dermatol..

[35] Marcelo C.L., Tomich J. (1983). Cyclic AMP, Glucocorticoid, and Retinoid Modulation of in Vitro Keratinocyte Growth. J. Investig. Dermatol..

[36] Halprin K.M., Adachi K., Yoshikawa K., Levine V., Mui M.M., Hsia S.L. (1975). Cyclic Amp And Psoriasis. J. Investig. Dermatol..

[37] Billi A.C., Gudjonsson J.E., Voorhees J.J. (2019). Psoriasis: Past, Present, and Future. J. Investig. Dermatol..

[38] Cortez Ghio S., Cantin-Warren L., Guignard R., Larouche D., Germain L. (2018). Are the Effects of the Cholera Toxin and Isoproterenol on Human Keratinocytes’ Proliferative Potential Dependent on Whether They Are Co-Cultured with Human or Murine Fibroblast Feeder Layers?. Int. J. Mol. Sci..

[39] Bullock A.J., Higham M.C., MacNeil S. (2006). Use of Human Fibroblasts in the Development of a Xenobiotic-Free Culture and Delivery System for Human Keratinocytes. Tissue Eng..

[40] Bharati K., Ganguly N.K. (2011). Cholera toxin: A paradigm of a multifunctional protein. Indian J. Med. Res..

[41] Archer C.B., Hanson J.M., Morley J., Macdonald D.M. (1984). Mononuclear Leukocyte Cyclic Adenosine Monophosphate Responses in Psoriasis Are Normal. J. Investig. Dermatol..

[42] Iizuka H., Adachi K., Halprin K.M., Levine V. (1978). Cyclic Amp Accumulation in Psoriatic Skin: Differential Responses to Histamine, Amp, and Epinephrine by the Uninvolved and Involved Epidermis. J. Investig. Dermatol..

[43] Yoshikawa K., Adachi K., Halprin K.M., Levine V. (1975). On the lack of response to catecholamine stimulation by the adenyl cyclase system in psoriatic lesions*. Br. J. Dermatol..

[44] Lanna C., Cesaroni G.M., Mazzilli S., Bianchi L., Campione E. (2019). Small Molecules, Big Promises: Improvement of Psoriasis Severity and Glucidic Markers with Apremilast: A Case Report. Diabetes Metab. Syndr. Obes. Targets Ther..

[45] Steinkraus V., Steinfath M., Stove L., Korner C., Abeck D., Mensing H. (1993). β-Adrenergic receptors in psoriasis: Evidence for down-regulation in lesional skin. Arch. Dermatol. Res..

[46] Das N.S., Chowdary T.N., Sobhanadri C., Rao K.V. (1978). The effect of topical isoprenaline on psoriatic skin. Br. J. Dermatol..

[47] Pierre S., Eschenhagen T., Geisslinger G., Scholich K. (2009). Capturing adenylyl cyclases as potential drug targets. Nat. Rev. Drug Discov..

[48] Pálvölgyi A., Simpson J., Bodnár I., Bíró J., Palkovits M., Radovits T., Skehel P., Antoni F.A. (2018). Auto-inhibition of adenylyl cyclase 9 (AC9) by an isoform-specific motif in the carboxyl-terminal region. Cell. Signal..

[49] Tsuge K., Inazumi T., Shimamoto A., Sugimoto Y. (2019). Molecular mechanisms underlying prostaglandin E2-exacerbated inflammation and immune diseases. Int. Immunol..

[50] Shi Q., Yin Z., Zhao B., Sun F., Yu H., Yin X., Zhang L., Wang S. (2015). PGE2 Elevates IL-23 Production in Human Dendritic Cells via a cAMP Dependent Pathway. Mediat. Inflamm..

[51] Litvinov I.V., Bizet A.A., Binamer Y.M., Sasseville D., Jones D.A., Philip A. (2011). CD109 release from the cell surface in human keratinocytes regulates TGF-β receptor expression, TGF-β signalling and STAT3 activation: Relevance to psoriasis. Exp. Dermatol..

[52] Funding A.T., Johansen C., Kragballe K., Iversen L. (2007). Mitogen- and Stress-Activated Protein Kinase 2 and Cyclic AMP Response Element Binding Protein are Activated in Lesional Psoriatic Epidermis. J. Investig. Dermatol..

[53] Germain L., Rouabhia M., Guignard R., Carrier L., Bouvard V., Auger F.A. (1993). Improvement of human keratinocyte isolation and culture using thermolysin. Burns.

[54] Rioux G., Pouliot-Bérubé C., Simard M., Benhassine M., Soucy J., Guérin S.L., Pouliot R. (2018). The Tissue-Engineered Human Psoriatic Skin Substitute: A Valuable In Vitro Model to Identify Genes with Altered Expression in Lesional Psoriasis. Int. J. Mol. Sci..

