# Supplementary material for: A Tissue-Engineered Human Psoriatic Skin Model to Investigate the Implication of cAMP in Psoriasis: Differential Impacts of Cholera Toxin and Isoproterenol on cAMP Levels of the Epidermis
Source: Int J Mol Sci. 2020 Jul 23;21(15):5215. doi: 10.3390/ijms21155215 (PMC7432929; doi:10.3390/ijms21155215)
Supplement: Supplementary file 1 [file ijms-21-05215-s001.pdf]

**SUPPLEMENTARY MATERIAL**

**Figure**

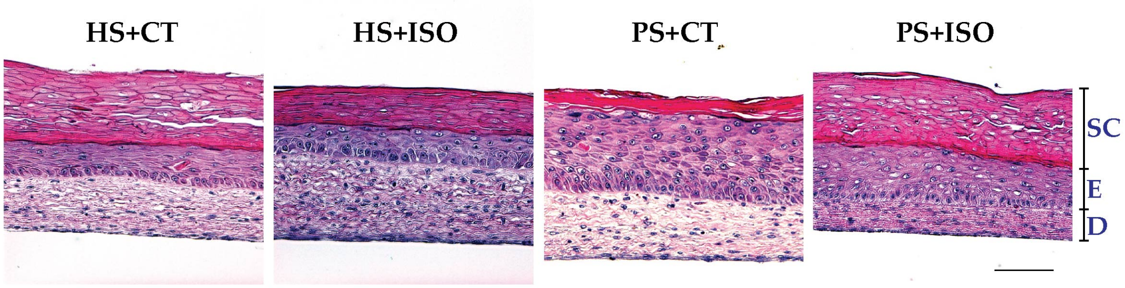


**Figure S1**. Hematoxylin and eosin staining of the skin substitutes presented in fig. 2 (panel e-h) (D: Dermis, E: Epidermis living layers, SC: *Stratum corneum*). Scale bar: 100 µm.
